# Supplementary material for: The NE/AAT/CBG axis regulates adipose tissue glucocorticoid exposure
Source: Nat Commun. 2025 Jan 9;16:545. doi: 10.1038/s41467-024-55693-x (PMC11718191; doi:10.1038/s41467-024-55693-x)
Supplement: Supplementary file 1 — Supplementary Information [file 41467_2024_55693_MOESM1_ESM.pdf]

Supplementary Figure 1.

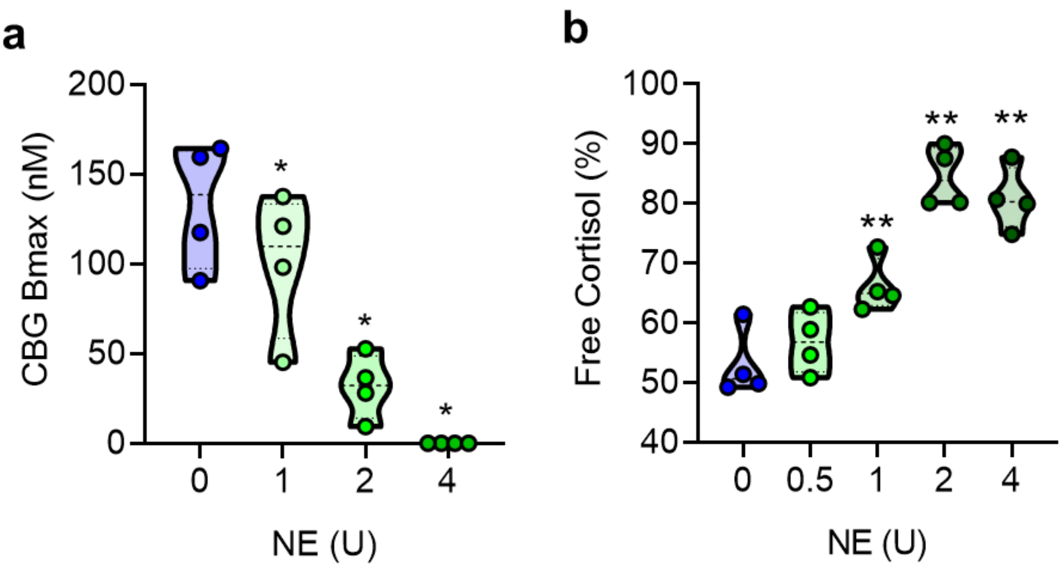

a-b, Dose response to NE treatment for human serum CBG binding capacity (a) and free cortisol (b). Note free cortisol (%) values do not reflect physiological levels as serum was diluted. Data are presented as mean (dashed line) -/+ SD (dotted line). *N = 4* per group. Comparisons are by One-way ANOVA; *P<0.05, **P<0.01 vs Vehicle (0).

8 Supplementary Figure 2.

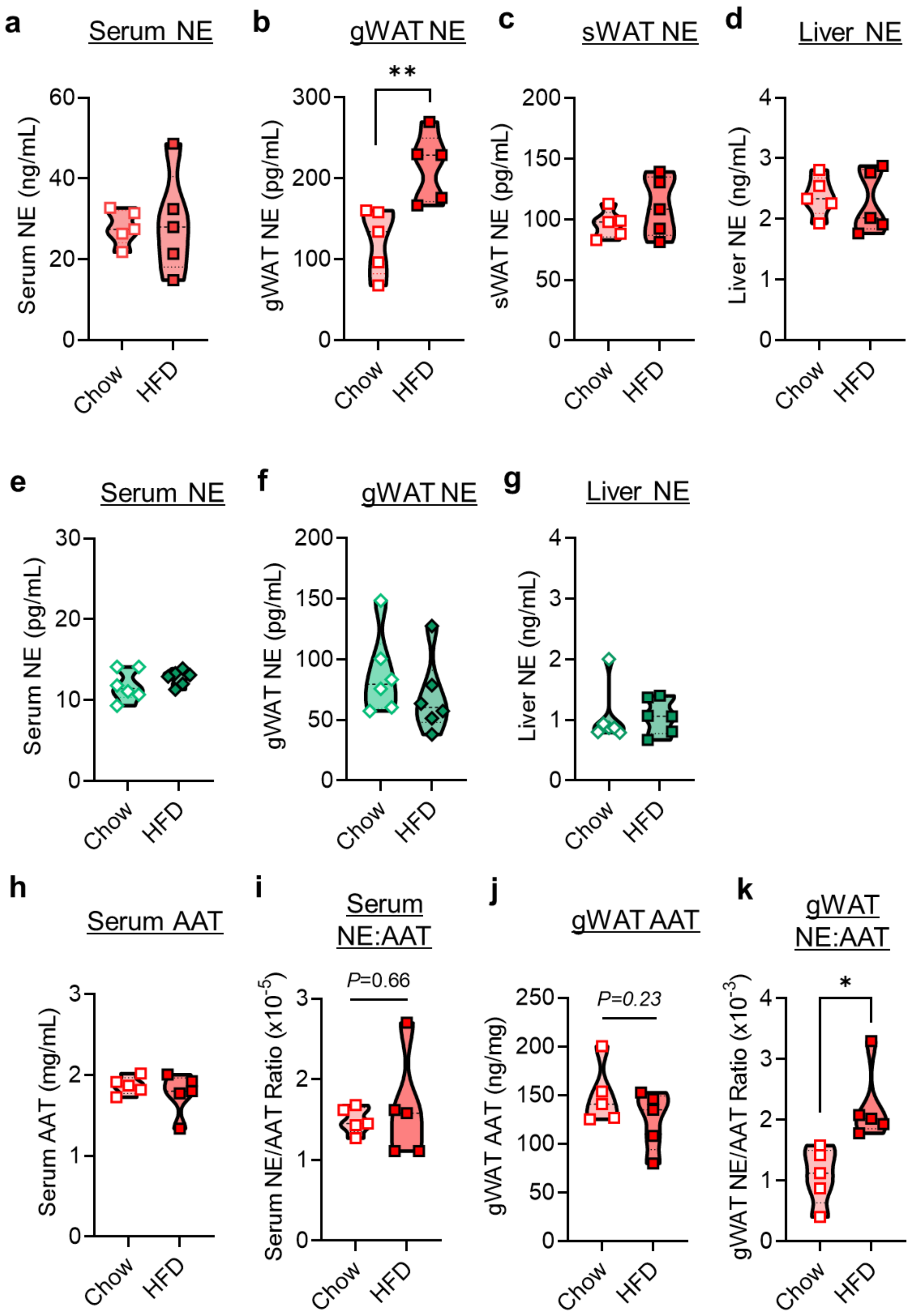

a, Serum NE levels in male C57Bl/6J mice fed a high fat diet (HFD; 58% kcal fat with sucrose) or regular rodent chow (chow) for 8 weeks. **b-d,** Tissue NE levels were quantified in visceral gonadal adipose (gWAT) (b), subcutaneous adipose (sWAT) (c), and liver (d) from male mice with indicated diet. **e**, Serum NE levels in female C57Bl/6J mice fed a high fat diet (HFD; 58% kcal fat with sucrose) or regular rodent chow (chow) for 8 weeks. **f-g,** Tissue NE levels were quantified in gWAT and liver from female mice with indicated diet. **h-i,** Serum AAT levels and calculated NE:AAT ratio from male mice with indicated diet. **j-k,** gWAT AAT levels and calculated NE:AAT ratio from male mice wih indicated diet. Data are presented as mean (dashed line) -/+ SD (dotted line). N=5 per group for males, n=6 per group for females. Comparisons were by two-tailed unpaired t-test; *$P<0.05$, **$P<0.01$.

Supplementary Figure 3.

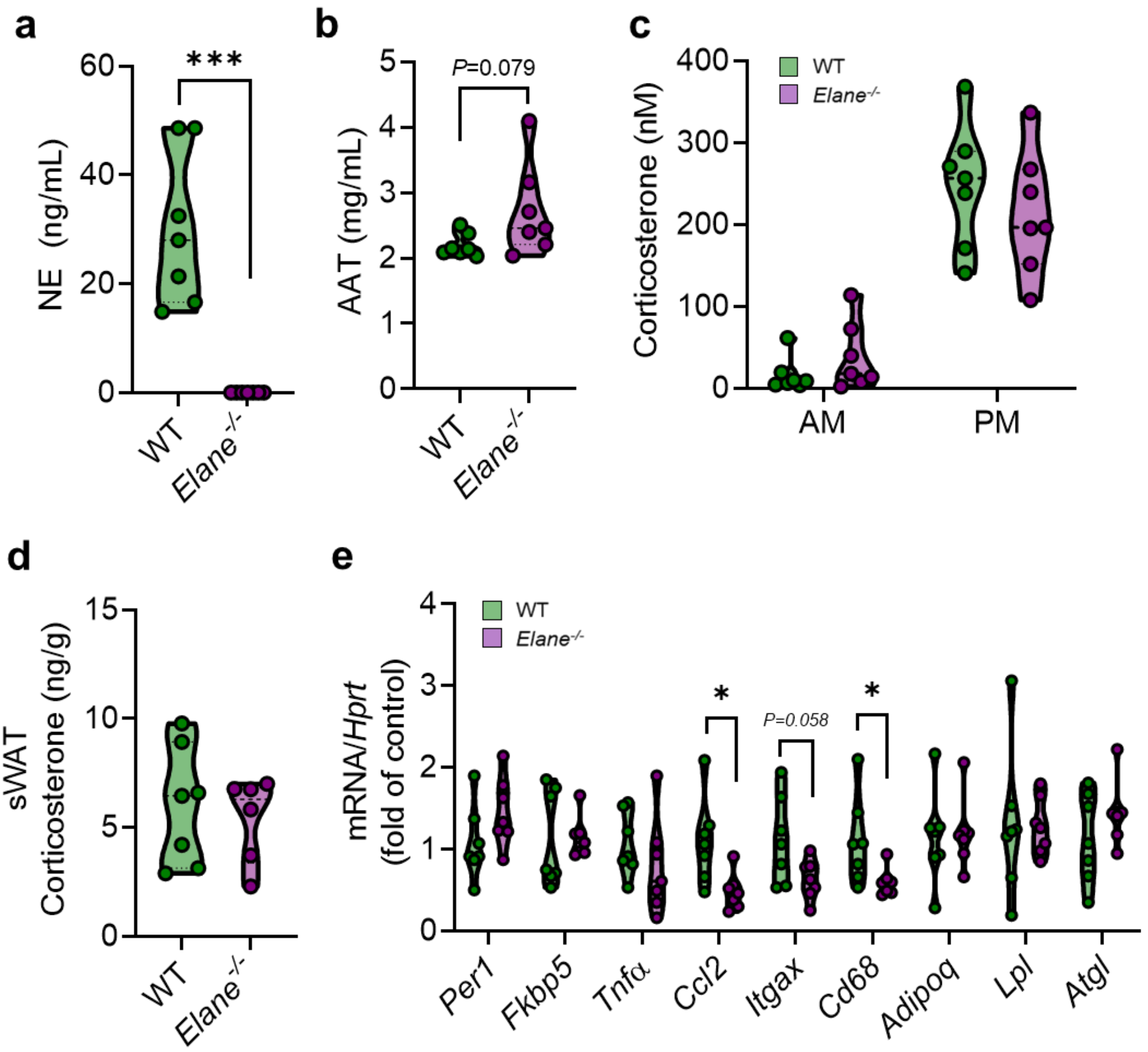

a-b, Serum profile of male *Elane*$^{-/-}$ and WT mice fed HFD for 8 weeks, including NE (a) and AAT (b). **c,** Diurnal serum corticosterone measured after 7 weeks of diet at 7am (AM) and 7pm (PM). **d,** Subcutaneous adipose (sWAT) corticosterone levels in mice with indicated genotypes. **e,** mRNA transcript expression in sWAT from mice with indicated genotypes. Data are presented as mean (dashed line) -/+ SD (dotted line). WT *n* = 7, *Elane*$^{-/-}$ *n* = 7. Data are analysed by two-tailed unpaired t-test. *P<0.05, ***P<0.001.

Supplementary Figure 4.

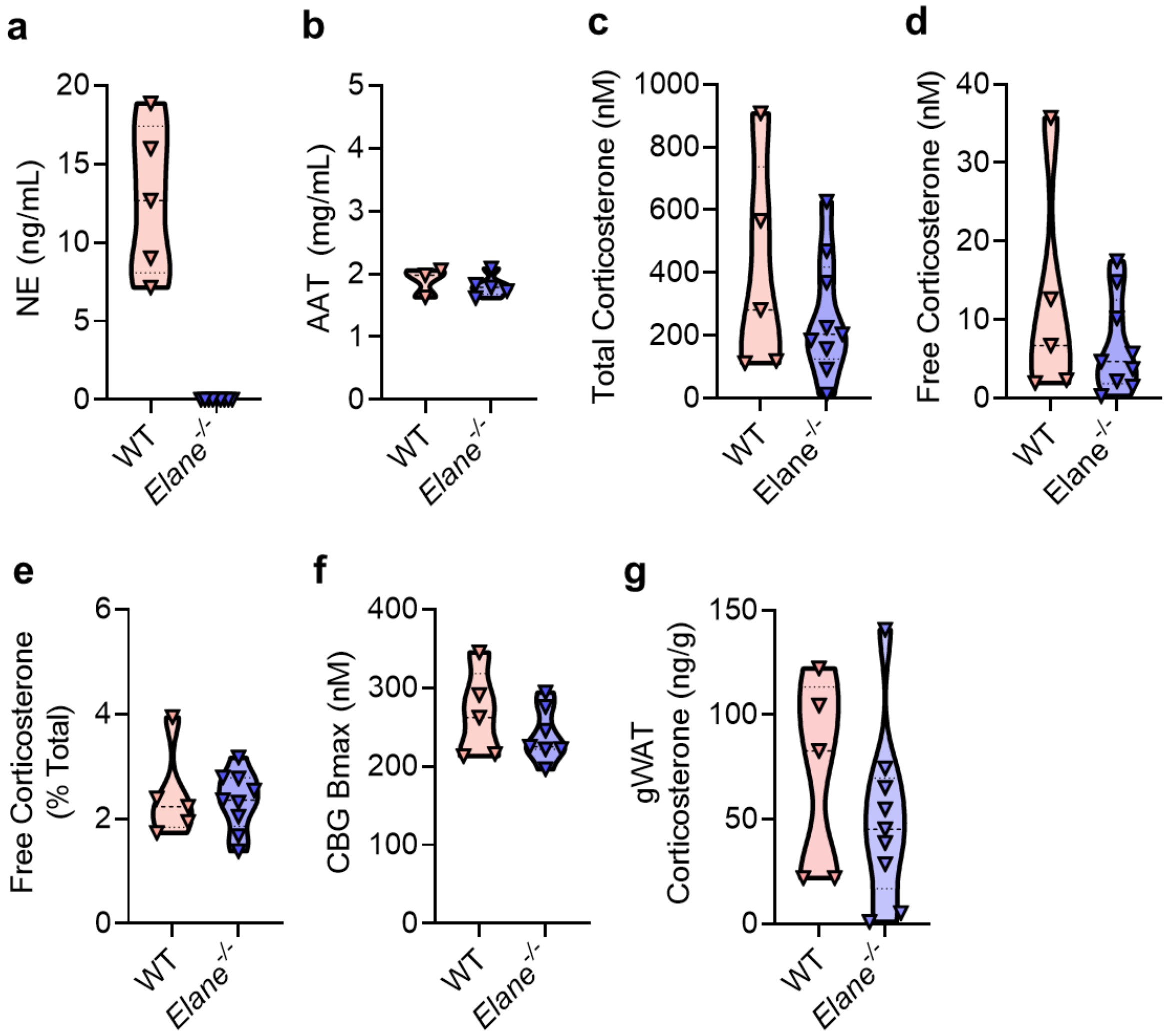

a-f, Serum profile of female *Elane*$^{-/-}$ and WT mice fed HFD for 8 weeks, including NE (a), AAT (b), total corticosterone (c), free corticosterone (d), % free corticosterone (e), and CBG binding capacity (f). **g,** Corticosterone levels in visceral gonadal adipose (gWAT) from mice with indicated genotypes. Data are presented as mean (dashed line) -/+ SD (dotted line). WT *n* = 5, *Elane*$^{-/-}$ *n* = 9 (For AAT, WT *n* = 3, ELA-KO *n* = 5). Data are analysed by two-tailed unpaired t-test .

Supplementary Figure 5.

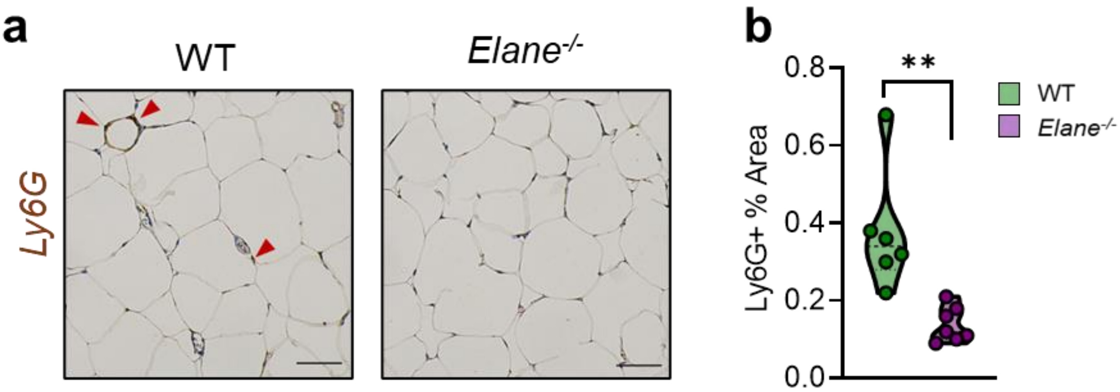

a-b, gWAT from male WT and *Elane*$^{-/-}$ mice fed HFD for 8 weeks stained with neutrophil-specific Ly6G by IHC and quantified. Red arrows indicate neutrophils (positive Ly6G staining). Scale bar = 40µm. Data are presented as mean (dashed line) -/+ SD (dotted line). WT *n* = 6*, Elane*$^{-/-}$ *n* = 7. Data are analysed by two-tailed unpaired t-test *P<0.05.

Supplementary Figure 6.

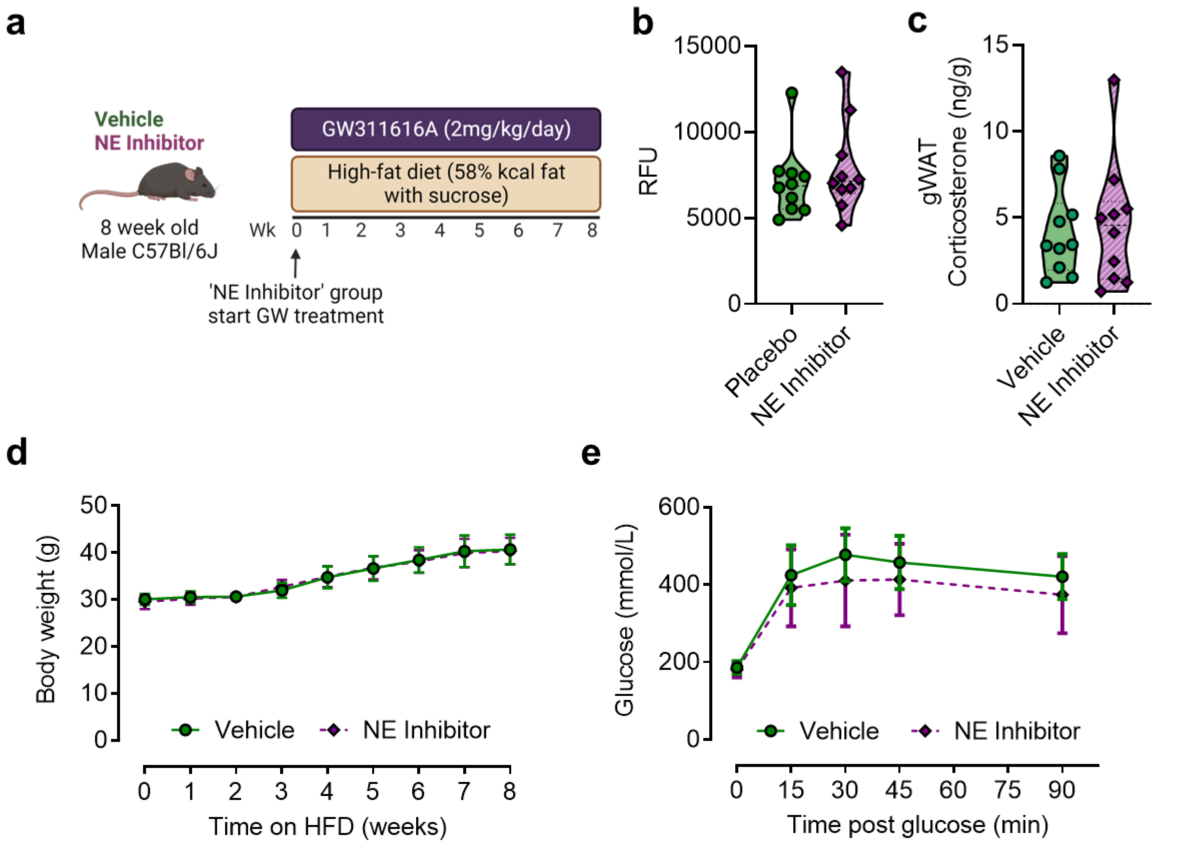

a, study design indicating adult (8 week old) male C57Bl6/J mice were fed a high-fat diet (58% kcal fat with sucrose) and co-administered the NE inhibitor (GW311616A; 2mg/kg) or vehicle (H_2O) every other day by oral gavage for 8 weeks. Created in BioRender (https://BioRender.com/k10s703). **b**, serum NE activity at cull (8 weeks) was unchanged between mice receiving NE inhibitor (purple diamond) or Vehicle (green circle). **c**, corticosterone levels in gWAT were unchanged between groups. **d**, body weights measured weekly over the course of treatment showed no change, and **e**, glucose tolerance tests performed between week 7 and 8 also showed no change between groups. Data are presented as mean -/+ SD. N=10 per group. Data were analysed by two-tailed unpaired t-test or RM Two-way ANOVA.

Supplementary Figure 7.

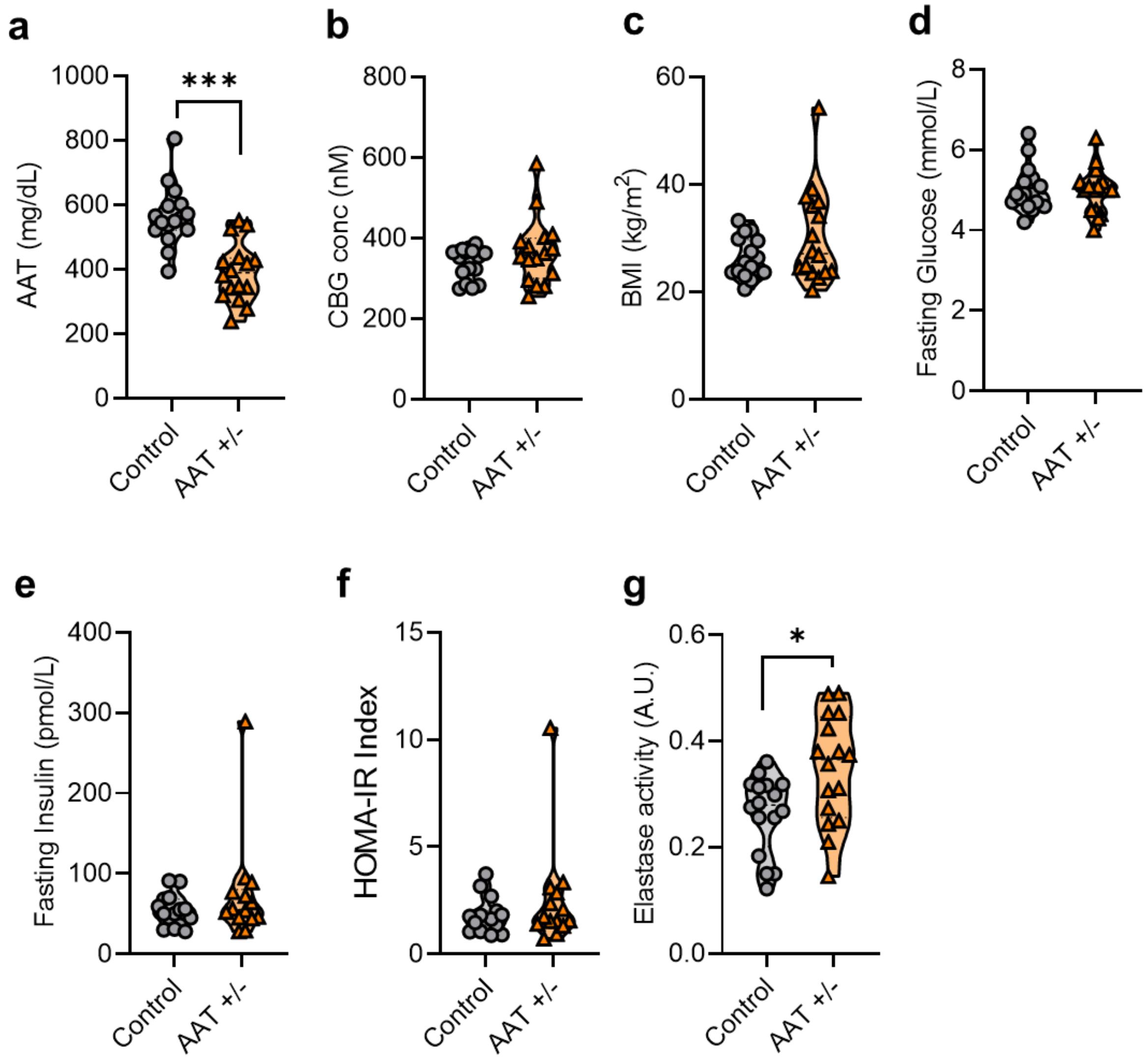

Baseline characteristics of participants with heterozygous mutations in SERPINA1 and matched controls. **a,** Serum, c alpha-1 antitrypsin (AAT). **b,** serum CBG. **c**, BMI. **d**, Fasting blood glucose. **e**, Fasting blood insulin. **f**, HOMA-IR. **g**, Serum elastase activity. Data are presented mean -/+ SD. Control *n = 16,* AAT+/- *n = 16* (For Fasting Insulin and HOMA-IR, Control *n = 15*). Data are analysed by two-tailed unpaired t-tests. *P<0.05, ***P<0.001.

Supplementary Figure 8.

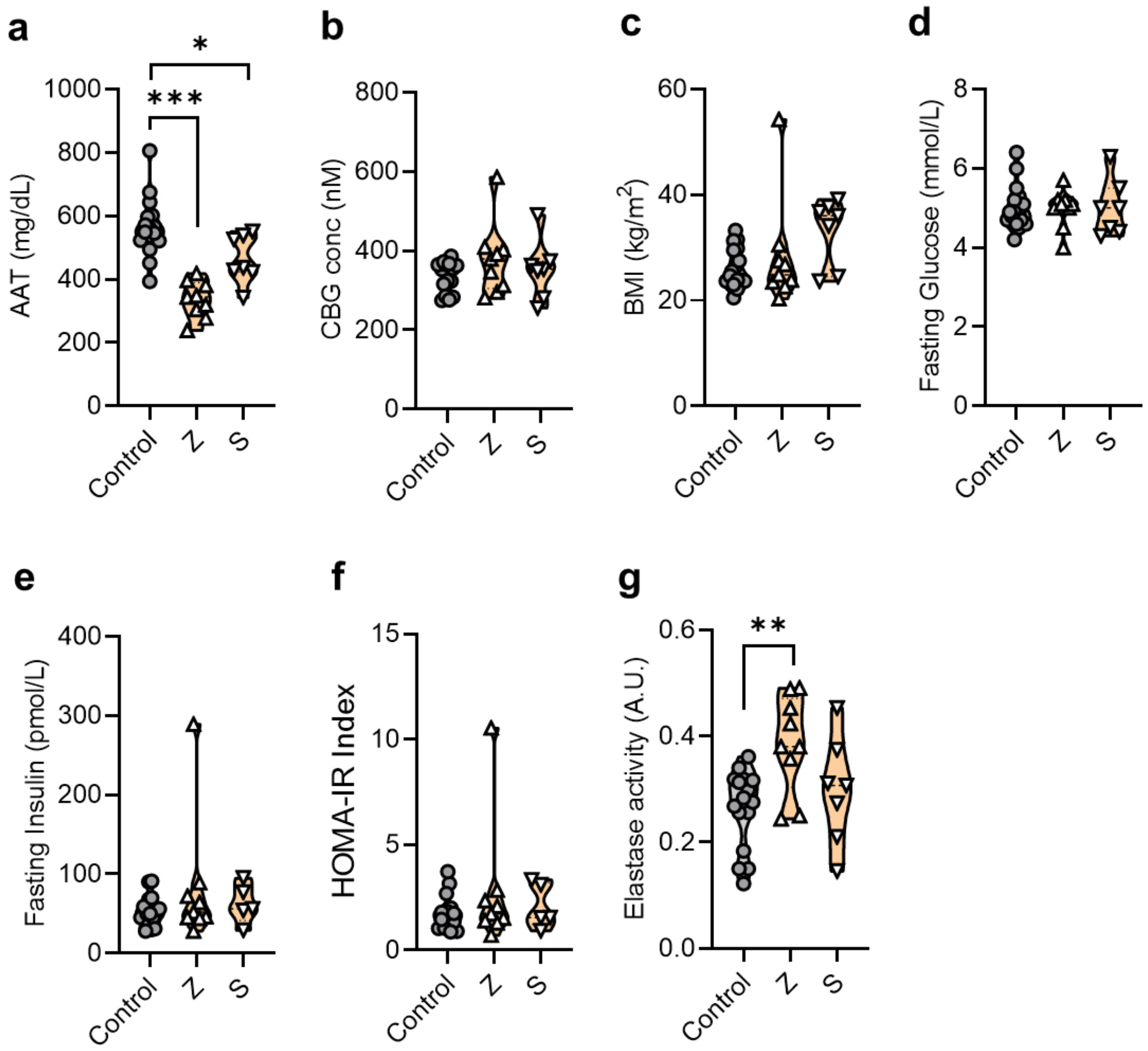

Baseline characteristics, presented by *SERPINA1* allele, of participants with heterozygous mutations in *SERPINA1* (‘Z’ = PiMZ, ‘S’ = PiMS) and matched controls. **a,** Serum, c alpha-1 antitrypsin (AAT). **b,** serum CBG. **c**, BMI. **d**, Fasting blood glucose. **e**, Fasting blood insulin. **f**, HOMA-IR. **g**, Serum elastase activity. Data are presented mean -/+ SD. Control *n = 16,* ‘Z’ *n = 9,* ‘S’ *n = 7* (For Fasting Insulin and HOMA-IR, Control *n = 15*)*.* Data are analysed by one-way ANOVA (with Tukey’s post hoc tests as appropriate). *P<0.05, P<0.01, ***P<0.001.

Supplementary Figure 9.

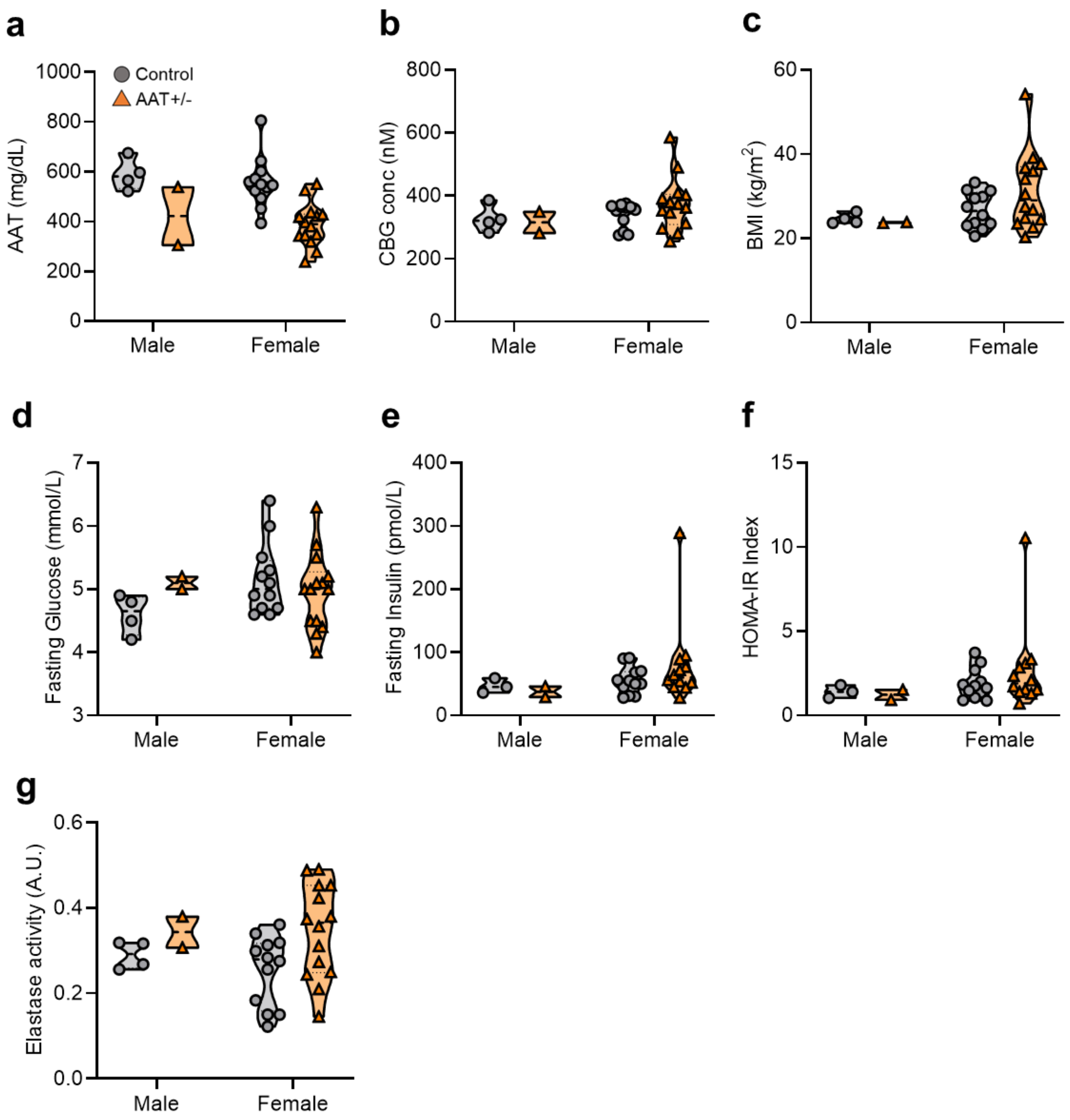

Baseline characteristics, presented by Sex, of participants with heterozygous mutations in *SERPINA1* (orange triangles) and matched controls (grey circles). **a,** Serum AAT. **b,** Serum CBG. **c**, BMI. **d**, Fasting blood glucose. **e**, Fasting insulin. **f**, HOMA-IR. **g**, Serum elastase activity. Data are presented mean -/+ SD. Male Control *n = 4,* Male AAT+/- *n = 2,* Female Control *n = 12,* Female AAT +/- *n = 14.* (For Fasting Insulin and HOMA-IR, Male Control *n* = *3*).

Supplementary Figure 10.

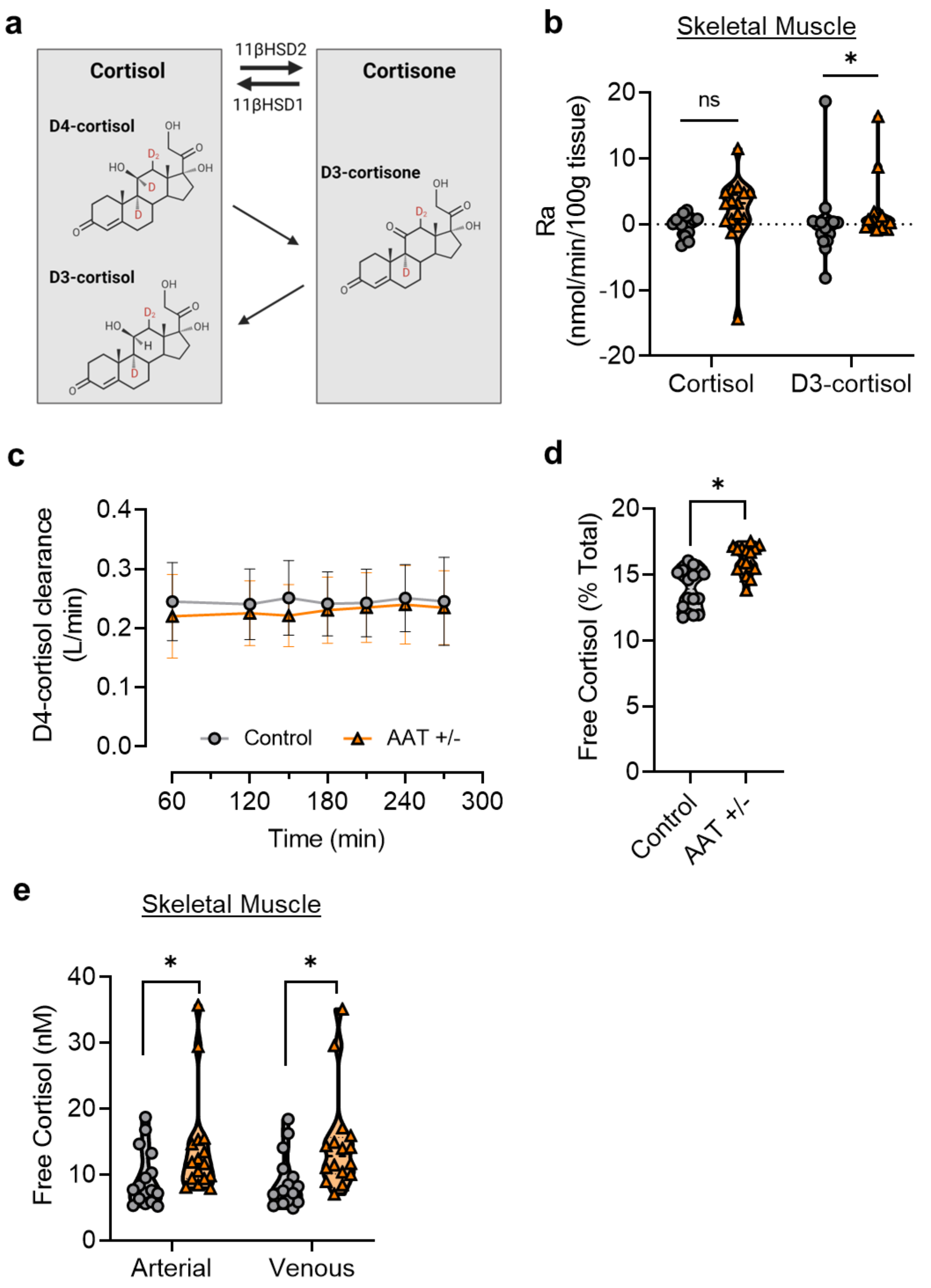

a, Overview of interconversion between D4-cortisol, D3-cortisone, and D3-cortisol, mediated by the glucocorticoid metabolising enzymes 11βHSD1/2. **b,** Rate of appearance (Ra) of cortisol and D3-cortisol across skeletal muscle in subjects with

heterozygous mutations in *SERPINA1* (AAT+/-) or controls (Control). **c,** Whole body D4-cortisol clearance in indicated genotypes. **d,** Arterialised percent free cortisol in indicated genotypes. **e,** Percent free cortisol across skeletal muscle. Data are presented mean -/+ SD. AAT+/- $n = 16$, Control $n = 16$. Data are analysed by RM two-way ANOVA (with Sidak's post-hoc tests as appropriate) or two-tailed unpaired t-tests. *$P<0.05$. ns = not significant.

Supplementary Figure 11.

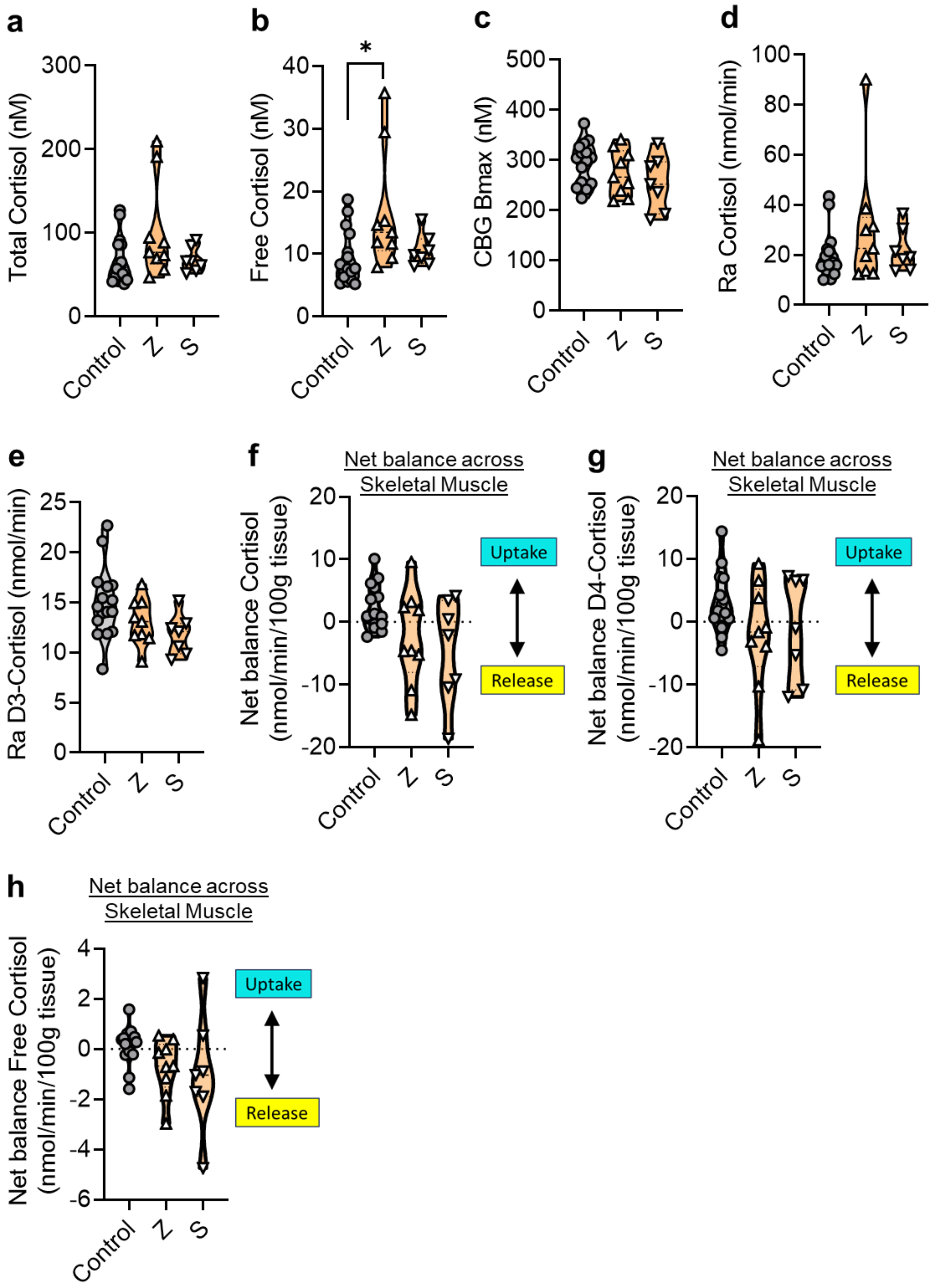

Plasma glucocorticoid profile during steady state D4-cortisol infusion (180 – 270 min), presented by *SERPINA1* allele, of participants with heterozygous mutations in *SERPINA1* (‘Z’ = PiMZ, ‘S’ = PiMS) and matched controls. **a**, free cortisol. **b**, total cortisol. **c**, CBG binding capacity. **d-e,** Whole body rate of appearance (Ra) of cortisol (d) and D3-cortisol (e) during steady state. **f,** Net balance of cortisol and **g**, D4-cortisol across skeletal muscle. **h,** Net balance of free cortisol across skeletal muscle. Data are presented as mean (dashed line) -/+ SD (dotted line). Control *n = 16,* AAT+/- *n = 16*. Data are analysed by one-way ANOVA (with Tukey’s post hoc tests as appropriate).

Supplementary Figure 12.

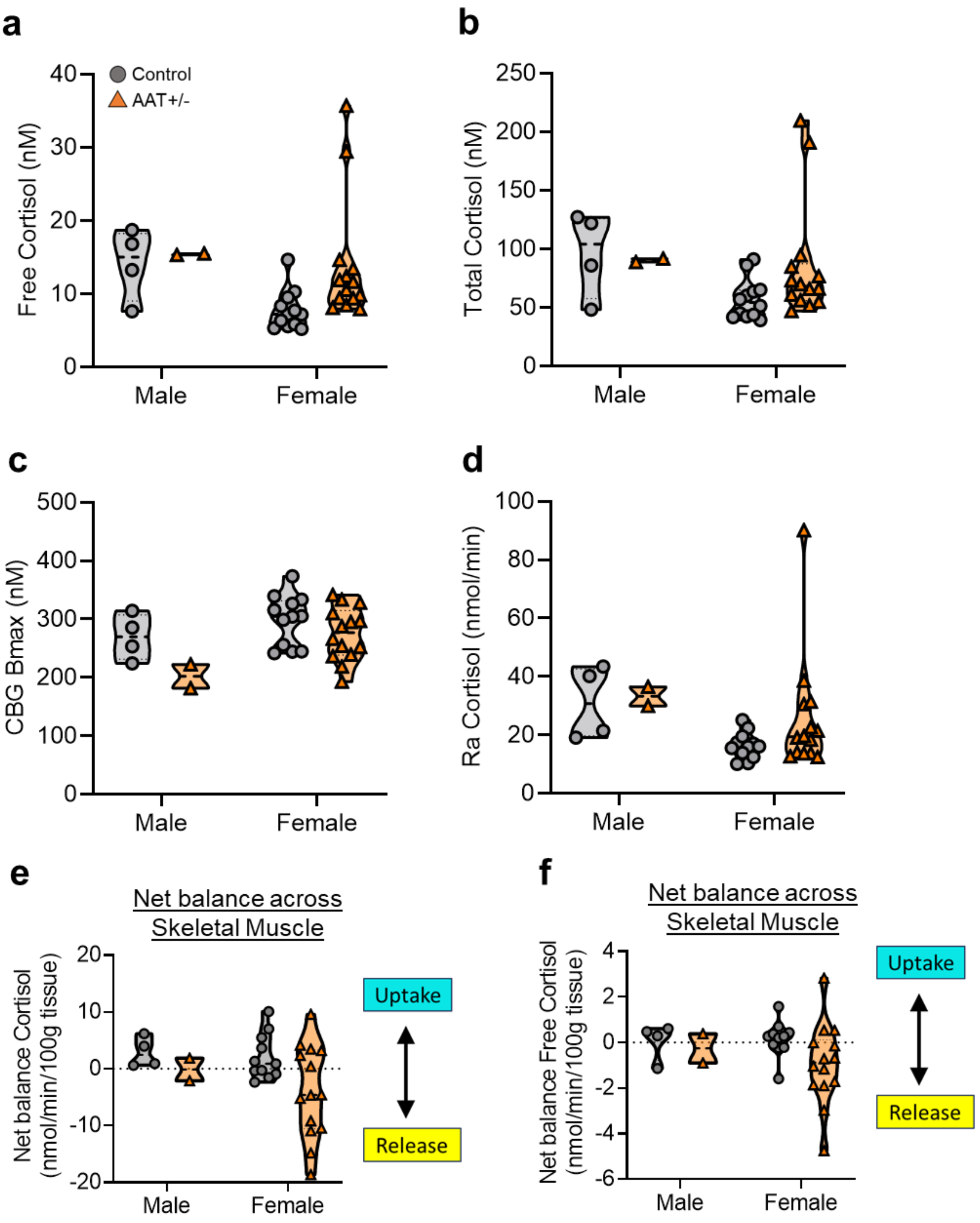

Plasma glucocorticoid profile during steady state D4-cortisol infusion (180 – 270 min), presented by Sex, of participants with heterozygous mutations in *SERPINA1* (orange triangles) and matched controls (grey circles). **a**, free cortisol. **b**, total cortisol. **c**, CBG

binding capacity. **d,** Whole body rate of appearance (Ra) of cortisol. **e,** Net balance of cortisol and **f,** free cortisol across skeletal muscle. Data are presented as mean (dashed line) -/+ SD (dotted line). Male Control *n = 4,* Male AAT+/- *n = 2,* Female Control *n = 12,* Female AAT +/- *n = 14.*

Supplementary Figure 13.

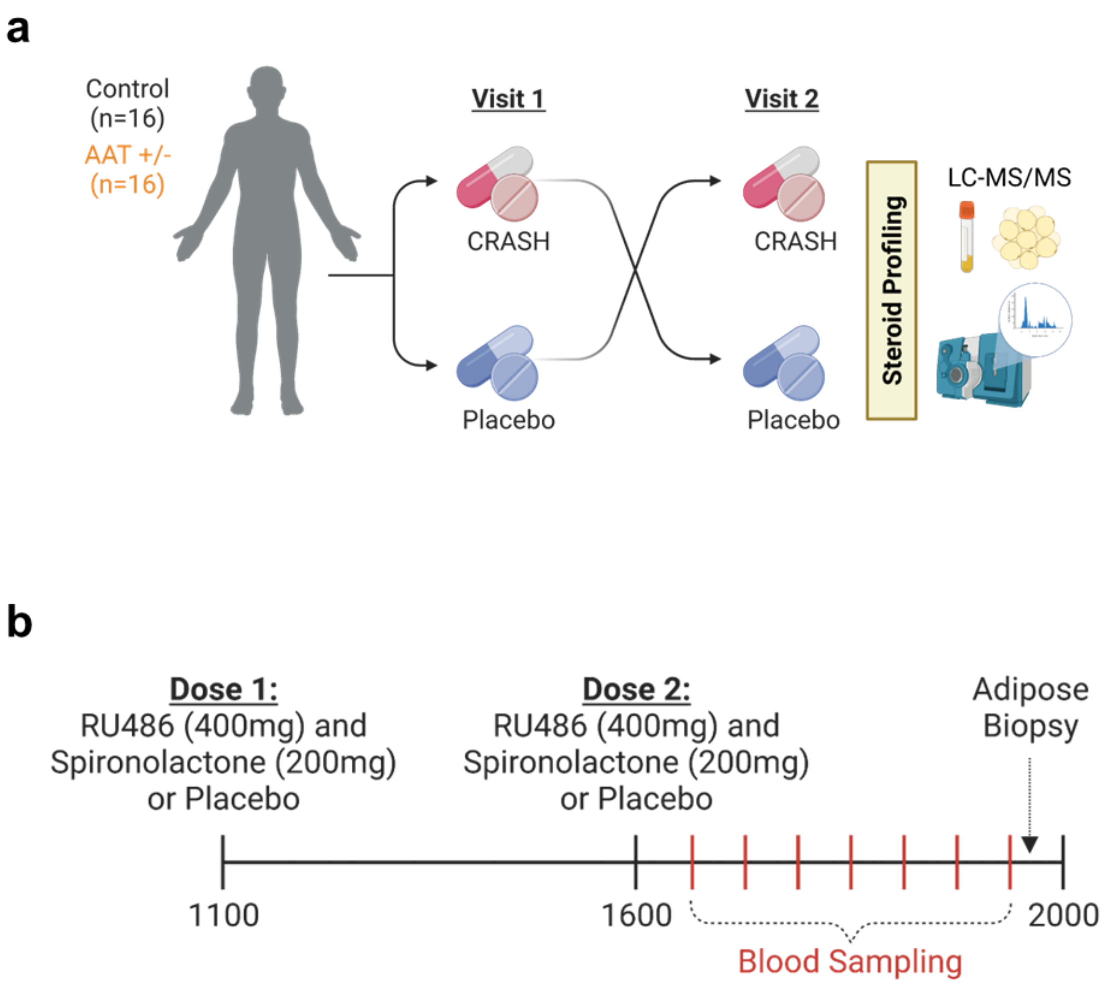

a, Overview of human study indicating crossover design and administration of combined RU486 and spironolactone (CRASH) or placebo in subjects with heterozygous mutations in *SERPINA1* (AAT+/-) or controls (Control). Created in BioRender (https://BioRender.com/y42q816). **b,** Overview of visit protocol, indicating time of treatment administration (1100 and 1600 h), blood sampling from anterograde 22G intravenous cannula inserted in arm was carried out every 30min post administration of Dose 2, up to +210 min. At +215 min, an adipose biopsy was performed, before participant was discharged. RU486 – mifepristone.

Supplementary Figure 14.

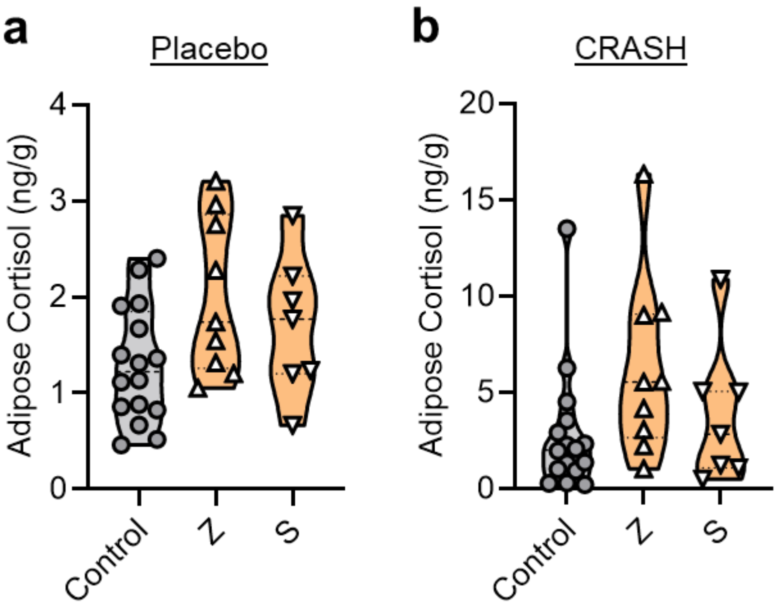

A randomised, double-blind crossover study, using either a combination of RU486 and spironolactone (CRASH) or placebo, presented by *SERPINA1* allele, in subjects with heterozygous mutations in *SERPINA1* (‘Z’ = PiMZ, ‘S’ = PiMS) and matched controls (Control). **a,** Cortisol levels in subcutaneous abdominal adipose from subjects undergoing Placebo phase. **b,** Cortisol levels in subcutaneous abdominal adipose from subjects undergoing ‘CRASH’ phase. Data are presented as mean (dashed line) -/+ SD (dotted line). Control: Placebo *n = 16,* AAT+/-: Placebo *n = 16,* Control: CRASH *n = 16,* AAT+/-: CRASH *n = 16*. Data are analysed by one-way ANOVA.

Supplementary Figure 15.

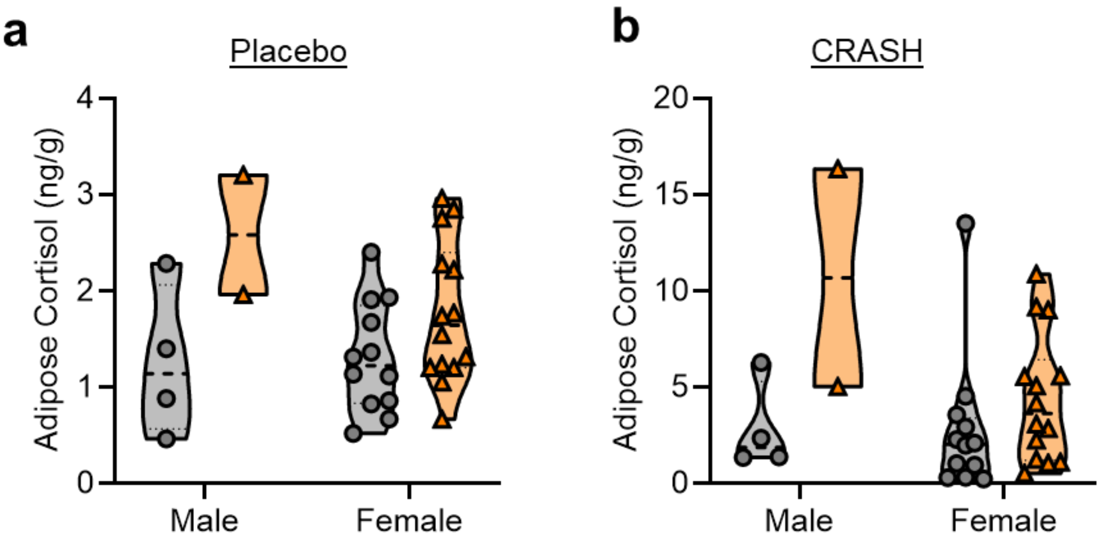

A randomised, double-blind crossover study, using either a combination of RU486 and spironolactone (CRASH) or placebo, presented by Sex, in subjects with heterozygous mutations in *SERPINA1* (orange triangles) and matched controls (grey circles). **a,** Cortisol levels in subcutaneous abdominal adipose from subjects undergoing Placebo phase. **b,** Cortisol levels in subcutaneous abdominal adipose from subjects undergoing 'CRASH' phase. Data are presented as mean (dashed line) -/+ SD (dotted line). Male Control *n = 4,* Male AAT+/- *n = 2,* Female Control *n = 12,* Female AAT +/- *n = 14.*

Supplementary Table 1

Predictor Variable	Fitted Regression Model	Overall Regression	P-Value
BMI (kg/m^2)	Y = 0.1713*X + 25.75	R^2 = .002, F(1, 30) = 0.054	*0.817*
Fasting insulin (pmol/L)	Y = 3.069*X + 16.19	R^2 = .013, F(1, 29) = 0.358	*0.554*
AAT (mg/dL)	Y = -26.92*X + 892.2	R^2 = .136, F(1, 30) = 4.722	***0.038***
CBG Bmax (nM)	Y = -13.87*X + 486.8	R^2 = .261, F(1, 30) = 10.58	***0.003***

Linear regression was used to test if predictor variables including BMI, fasting insulin, AAT levels, or CBG Bmax significantly predicted percent free cortisol in human subjects. The fitted regression model = regression equation, where Y = percent free cortisol, and X = Predictor Variable.

Supplementary Table 2

Measurement	Control		AAT+/-	
	Male	Female	Male	Female
n number	4	12	2	14
Total Cortisol (nM)	128 (42)	107 (35)	131 (26)	122 (35)
Free Cortisol (%)	13.4 (1.4)	12.9 (1.5)	15.8 (1.0)	15.1 (0.7)
CBG (nM)	358 (29)	361 (47)	313 (3)	380 (65)
Adipose Cortisol (ng/g)	1.3 (0.7)	1.3 (0.6)	3.2 (0.4)	1.8 (0.7)

Glucocorticoid profile in subjects with heterozygous mutations in *SERPINA1* (AAT+/) and matched controls (Control) when sub-categorised by sex. Circulating data (total cortisol, free cortisol fraction, CBG) represent average across study period during placebo phase. Adipose cortisol from placebo phase in indicated genotypes and sex. Data are mean (-/+ SD).

Supplementary Table 3

Steroid	Internal Standard	Q1 Mass *(m/z)*	Q3 Mass *(m/z)*	DP (V)	CE (V)	CXP (V)	Time (min)
A 1	Epi-F	345.1	121.0	66	31	12	3.6
A 2		345.1	91.2	66	83	40	3.6
B 1	D8-B	347.1	121.1	76	29	8	5.3
B 2		347.1	90.9	76	75	12	5.3
E 1	Epi-F	361.1	163.1	81	31	26	2.9
E 2		361.1	77.1	81	107	10	2.9
F 1	Epi-F	363.1	121.2	76	31	8	3.5
F 2		363.1	91.1	76	83	10	3.5
**Internal Standards and tracers**							
d4-cortisol		367.3	121.1	76	31	8	3.4
Epi-F 1		363.1	121.2	76	31	8	3.1
Epi-F 2		363.1	91.1	76	83	10	3.1
d8-corticosterone		355.3	125.1	76	29	8	5.2

Positive ion multiple reaction monitoring (MRM) parameters and retention times for each steroid and internal standard, as analysed on a Kinetex C18 (150 x 2.1 mm; 2.6 μm) column on an Shimadzu Nexera X2 uHPLC and QTrap 6500+ mass spectrometer following electrospray ionisation. A - 11-dehydrocorticosterone; B - corticosterone; E - cortisone; F - cortisol; DP - Declustering Potential; CE - Collision Energy; CXP - Collision Cell Exit Potential; RT - Retention Time. Quantifier (1) and Qualifier (2), MRM indicated accordingly.

Supplementary Table 4

Mouse

Gene Name	Forward Primer 5' - 3'	Reverse Primer 5' - 3'
Adipoq	ATCTGGAGGTGGGAGACCAA	GGGCTATGGGTAGTTGCAGT
Ccl2	AGCTGTAGTTTTTGTCACCAAGC	GACCTTAGGGCAGATGCAGT
Fkbp5	CCAAACGAAGGAGCAACGGTAAAA	GAACACCACATCTCGGCAATCAAA
G6pc	TCTGTCCCGGATCTACCTTG	GAAAGTTTCAGCCACAGCAA
Hprt	TCCTCCTCAGACCGCTTTT	CCTGGTTCATCATCGCTAATC
Hsd11b1	GGTTTTCTTCGTGTGTCCTACA	CCCTGGAGCATTTCTGGTCTG
Itgax	TTGGAGCTTCCAGTAAAATATGC	TCTCCTTTTCTGAGGTTGAGAAG
Lpl	AGAGAGGACTCGGAGACGTG	GGAGTTGCACCTGTATGCCT
Pepck	TTGAACTGACAGACTCGCCCT	GATATGCCCATCCGAGTCATG
Per1	AACGGGATGTGTTTCGGGGTGC	AGGACCTCCTCTGATTCGGCAG
Pnpla2	GGAACCAAAGGACCTGATGACC	ACATCAGGCAGCCACTCCAACA
Pparg	AGGGCGATCTTGACAGGAAA	CGAAACTGGCACCCTTGAAA
Rna18s1	AAACGGCTACCACATCCAAG	CCTCCAATGGATCCTCGTTA
Serpina1	GGCTGACCTCTCCGGAAT	GTCAGCACGGCCTTATGC
Serpina6	GAATGAGACAAGCACAGTGAAGGT	CGCCGAATCACGAAAGTAACT
Sgk1	CTTGGGCTATCTGCACTCCC	GCCCAAAGTCAGTGAGGACG
Tnfa	ATCGGTCCCCAAAGGGATGA	GGTGGTTTGCTACGACGTG

Human

Gene Name	Forward Primer 5' - 3'	Reverse Primer 5' - 3'
ADIPOQ	GGTGAGAAGGGTGAGAAAGGA	TTCACCGATGTCTCCCTTAG
FKBP5	GGATATACGCCAACATGTTCAA	CCATTGCTTTATTGGCCTCT
LPL	ATGTGGCCCGGTTTATCA	CTGTATCCCAAGAGATGGACATT
PEPCK	CGAAAGCTCCCCAAGTACAA	GCTCTCTACTCGTGCCACATC
PER1	CTCTTCCACAGCTCCCTCA	CTTTGGATCGGCAGTGGT
PNPLA2	CTCCACCAACATCCACGAG	CCCTGCTTGCACATCTCTC
PPIA	ATGCTGGACCCAACACAAAT	TCTTTCACTTTGCCAAACACC
RNA18S1	CTTCCACAGGAGGCCTACAC	CGCAAAATATGCTGGAACTTT
SGK1	CTCCTATGCATGCAAACACC	CCAAGGTTGATTTGCTGAGAA

Forward and reverse primers for SybrGreen qPCR in mouse and human tissue samples.
