# Supplementary material for: The NE/AAT/CBG axis regulates adipose tissue glucocorticoid exposure
Source: Nat Commun. 2025 Jan 9;16:545. doi: 10.1038/s41467-024-55693-x (PMC11718191; doi:10.1038/s41467-024-55693-x)
Supplement: Supplementary file 3 — Reporting Summary [file 41467_2024_55693_MOESM3_ESM.pdf]

Corresponding author(s): Mark Nixon

Last updated by author(s): Nov 22, 2024

Reporting Summary

Nature Portfolio wishes to improve the reproducibility of the work that we publish. This form provides structure for consistency and transparency in reporting. For further information on Nature Portfolio policies, see our Editorial Policies and the Editorial Policy Checklist.

Statistics

For all statistical analyses, confirm that the following items are present in the figure legend, table legend, main text, or Methods section.

n/a	Confirmed	
☐	☒	The exact sample size (*n*) for each experimental group/condition, given as a discrete number and unit of measurement
☐	☒	A statement on whether measurements were taken from distinct samples or whether the same sample was measured repeatedly
☐	☒	The statistical test(s) used AND whether they are one- or two-sided<br>*Only common tests should be described solely by name; describe more complex techniques in the Methods section.*
☒	☐	A description of all covariates tested
☐	☒	A description of any assumptions or corrections, such as tests of normality and adjustment for multiple comparisons
☐	☒	A full description of the statistical parameters including central tendency (e.g. means) or other basic estimates (e.g. regression coefficient) AND variation (e.g. standard deviation) or associated estimates of uncertainty (e.g. confidence intervals)
☐	☒	For null hypothesis testing, the test statistic (e.g. *F*, *t*, *r*) with confidence intervals, effect sizes, degrees of freedom and *P* value noted<br>*Give P values as exact values whenever suitable.*
☒	☐	For Bayesian analysis, information on the choice of priors and Markov chain Monte Carlo settings
☒	☐	For hierarchical and complex designs, identification of the appropriate level for tests and full reporting of outcomes
☒	☐	Estimates of effect sizes (e.g. Cohen's *d*, Pearson's *r*), indicating how they were calculated

Our web collection on statistics for biologists contains articles on many of the points above.

Software and code

Policy information about availability of computer code

Data collection	N/A
Data analysis	Data analysis was performed in Microsoft Excel and GraphPad Prism v8.0.

For manuscripts utilizing custom algorithms or software that are central to the research but not yet described in published literature, software must be made available to editors and reviewers. We strongly encourage code deposition in a community repository (e.g. GitHub). See the Nature Portfolio guidelines for submitting code & software for further information.

Data

Policy information about availability of data

All manuscripts must include a data availability statement. This statement should provide the following information, where applicable:

- Accession codes, unique identifiers, or web links for publicly available datasets
- A description of any restrictions on data availability
- For clinical datasets or third party data, please ensure that the statement adheres to our policy

The source data underlying the figures in this manuscript are provided as a Source Data File.

Research involving human participants, their data, or biological material

Policy information about studies with human participants or human data. See also policy information about sex, gender (identity/presentation), and sexual orientation and race, ethnicity and racism.

Reporting on sex and gender	Sex/gender was not purely self-reported by subjects, but matched their unique Community Health Index (CHI) number which, in Scotland, acts as a record of date of birth and identifies each subject as male or female from birth. For all subjects, self-reported gender matched sex as determined by their CHI number. Sex was not considered in the study design. Disaggregated sex data has been provided in the Supplementary Information.
Reporting on race, ethnicity, or other socially relevant groupings	No socially relevant categorisations were used in this study.
Population characteristics	Participants were matched for age, BMI and sex. All participants completed a comprehensive screening evaluation that included a medical history and physical examination, and standard blood tests to determine eligibility. The following inclusion criteria were required: aged 18-70 years, asymptomatic carriers and non-carriers of AAT deficiency (age-, sex- and body mass index-matched controls), women of a childbearing potential who were willing to use a barrier method of contraception. Potential participants with abnormal screening bloods (full blood count and renal, liver and thyroid function) of clinical significance, those with active acute or chronic medical conditions requiring a therapy (including hormonal contraceptive use), those with history of oral, topical or inhalational corticosteroid use in the preceding six months, or those that were pregnant, seeking to become pregnant or lactating were excluded.
Recruitment	16 asymptomatic carriers (cases) of AAT deficiency alleles (genotypes PiMS (7 subjects) & PiMZ (9 subjects)) and 16 non-carriers (controls, genotype PiMM) were recruited in a 'first come' approach.
Ethics oversight	The study was approved by the Academic and Clinical Central Office for Research and Development (ACCORD) Medical Research Ethics Committee (AMREC) (reference 17-HV-032) and by NHS Lothian Research and Development (2017/0193). This study was classed as a 'Basic science research study involving procedures with human participants', and not as a clinical trial.

Note that full information on the approval of the study protocol must also be provided in the manuscript.

Field-specific reporting

Please select the one below that is the best fit for your research. If you are not sure, read the appropriate sections before making your selection.

☒ Life sciences ☐ Behavioural & social sciences ☐ Ecological, evolutionary & environmental sciences

For a reference copy of the document with all sections, see nature.com/documents/nr-reporting-summary-flat.pdf

Life sciences study design

All studies must disclose on these points even when the disclosure is negative.

Sample size	Animals: n our previously published work on tracer measurement in adipose (PMID: 27535620), the mean variability of corticosterone in adipose tissue was 21.8pg/mL. An n of 7 per group provides 80% power to detect a 20% difference in adipose corticosterone concentrations.<br>Humans: In our previously published work on tracer measurement in adipose (PMID: 22511204), the mean arterio-venous difference in D4-cortisol concentration across adipose tissue was 3.39 nmol/L, with a standard deviation of 0.95. An n of 16 per group provides 80% power to detect a 20% difference in subcutaneous adipose vein D4-cortisol concentration.
Data exclusions	No data were excluded
Replication	Experimental findings were from established techniques and analyses to ensure reproducibility.
Randomization	Animals: Mice were randomly allocated to dietary treatment/surgery prior to start of study.<br>Humans: Participants took either placebo or spironolactone 200mg/RU486 400mg, administered twice on study visit days. Participants were given placebo and spironolactone/RU486 in random order. Randomisation was undertaken by Tayside Pharmaceuticals and kept securely in a sealed envelope until all measurements were complete.
Blinding	Animals: Investigators were blinded to genotype (WT or Elane-/-) and surgery (group Sham or adrenalectomy) through use of a randomised ID 'Key' generated for each mouse. Investigators were blinded during analyses.<br>Humans: Investigators were blinded to the treatment phase in the HPA axis study. Participants took either placebo or spironolactone 200mg/RU486 400mg, administered twice on study visit days (as outlined in 'Randomization' above. Investigators were blinded to the treatment group, with only clinical research facility staff being unblinded. Investigators were blinded during analyses.

Reporting for specific materials, systems and methods

We require information from authors about some types of materials, experimental systems and methods used in many studies. Here, indicate whether each material, system or method listed is relevant to your study. If you are not sure if a list item applies to your research, read the appropriate section before selecting a response.

Materials & experimental systems

n/a	Involved in the study
☐	☒ Antibodies
☐	☒ Eukaryotic cell lines
☒	☐ Palaeontology and archaeology
☐	☒ Animals and other organisms
☒	☐ Clinical data
☒	☐ Dual use research of concern
☒	☐ Plants

Methods

n/a	Involved in the study
☒	☐ ChIP-seq
☒	☐ Flow cytometry
☒	☐ MRI-based neuroimaging

Antibodies

Antibodies used	Ly6G (neutrophil specific antibody; Rabbit mAB #87048, Cell Signaling)
Validation	IHC validation was performed on murine spleen samples (as per Cell Signaling instructions) using the Bond Rx (Lecia Biosystems)

Eukaryotic cell lines

Policy information about cell lines and Sex and Gender in Research

Cell line source(s)	Human embryonic kidney (HEK293) (from ATCC)
Authentication	Not authenticated
Mycoplasma contamination	Cell line routinely test negative for Mycoplasma contamination
Commonly misidentified lines (See ICLAC register)	None

Animals and other research organisms

Policy information about studies involving animals; ARRIVE guidelines recommended for reporting animal research, and Sex and Gender in Research

Laboratory animals	Mus musculus, C57Bl5/J, B6.129X1-Elanetm1Sds/J
Wild animals	Study did not involve wild animals
Reporting on sex	Both male and female mice were studied and are reported in the manuscript
Field-collected samples	Study did not involve samples collected from the field
Ethics oversight	University of Edinburgh Animal Welfare and Ethical Review Board

Note that full information on the approval of the study protocol must also be provided in the manuscript.

Plants

Seed stocks	*Report on the source of all seed stocks or other plant material used. If applicable, state the seed stock centre and catalogue number. If plant specimens were collected from the field, describe the collection location, date and sampling procedures.*
Novel plant genotypes	*Describe the methods by which all novel plant genotypes were produced. This includes those generated by transgenic approaches, gene editing, chemical/radiation-based mutagenesis and hybridization. For transgenic lines, describe the transformation method, the number of independent lines analyzed and the generation upon which experiments were performed. For gene-edited lines, describe the editor used, the endogenous sequence targeted for editing, the targeting guide RNA sequence (if applicable) and how the editor was applied.*
Authentication	*Describe any authentication procedures for each seed stock used or novel genotype generated. Describe any experiments used to assess the effect of a mutation and, where applicable, how potential secondary effects (e.g. second site T-DNA insertions, mosiacism, off-target gene editing) were examined.*
